# Clinical Characteristics of Paediatric RSV, Influenza, and SARS-CoV-2 Infections: Insights from Three Consecutive Seasons

**DOI:** 10.3390/v17111403

**Published:** 2025-10-22

**Authors:** Weronika Balas, Katarzyna Balas, Maria Gancarczyk, Joanna Kośka, Martyna Radelczuk, Agata Rosińska, Adam Sybilski

**Affiliations:** 1 Clinical Department of Paediatrics and Allergology, National Medical Institute of the Ministry of the Interior and Administration, 02-507 Warsaw, Poland; 2 Second Department of Paediatrics, Centre of Postgraduate Medical Education, 00-416 Warsaw, Poland

**Keywords:** fever, children, hypoxia, viruses, hospitalisation

## Abstract

Background: The purpose of this study was to retrospectively analyse the clinical presentation of RSV, Influenza, and SARS-CoV-2 infections in children across three consecutive seasons (2022/2023; 2023/2024; and 2024/2025). Methods: Of the 321 hospitalised patients, 129 (36%) tested positive for RSV, 110 (38%) for Influenza, and 82 (26%) for SARS-CoV-2. Children were aged ≤ 17 years (median: 15 months). The data were statistically analysed using the χ^2^ test, multinomial multivariable logistic regression, OR (odds ratio), and 95% CI (confidence interval). Results: Significant independent predictors of RSV infection were auscultatory abnormalities (OR: 15.9 [1.49–169]) and hospital admission ≥ 4 days after symptom onset (OR: 32.5 [1.19–907]). Among RSV-positive patients, compared with those aged < 6 months, those aged 7–24 months were more likely to present with higher CRP levels (OR 1.06 [1.003–1.13]), reduced appetite (OR 6.7 [1.62–27.67]), and longer duration of fever (OR 7.22 [1.47–35.59]), while in children > 24 months, only a longer duration of fever remained significant (OR 16.82 [2.14–162.4]). In Influenza, reduced appetite was the only characteristic feature in the 7–24-month age group (OR 13.55 [1.79–102.81]). In COVID-19, children aged 7–24 months more frequently had higher CRP levels (OR 1.108 [1.001–1.226]) and chronic diseases (OR 7.59 [1.115–51.64]), whereas in those >24 months, only CRP was significant (OR 1.16 [1.047–1.31]). Conclusions: RSV was associated with severe respiratory manifestations and later hospital admission, whereas Influenza and SARS-CoV-2 were characterised by milder courses with predominant upper respiratory symptoms. Observed age- and virus-specific patterns highlight the importance of continued surveillance and comparative research on major respiratory viruses in children.

## 1. Introduction

RSV, SARS-CoV-2, and Influenza virus cause mild, moderate, and severe viral respiratory infections. SARS-CoV2 infection is mild and mainly characterised by fever and respiratory symptoms, but it can rarely develop into a severe multisystem inflammatory syndrome in children (MIS-C) [1]. Meta-analysis from 2022 revealed that 2.41% of SARS-CoV-2-infected children died from this syndrome [2]. The SARS-CoV2 pandemic has influenced the epidemiology of Influenza and RSV: off-season increase, shift in the median age of onset, and more severe course of the disease [3]. Many articles report the differences in the clinical picture depending on the season and strain of the virus [4,5].

Since the 2023/2024 season, Influenza virus activity has returned to almost pre-pandemic levels in European countries [6]. Influenza commonly infects children, as shown by the meta-analyses of randomised studies: 32% of children under 6 years of age, 10.5% of school-age children, and only 2.5% of adults [7]. Influenza may cause pandemics and various complications, such as pneumonia, acute otitis media, neurological complications, myocarditis, and the exacerbation of existing chronic diseases.

RSV is a common seasonal pathogen in young children, which causes enormous morbidity and mortality [8]. The U.S. Centres for Disease Control and Prevention (CDC) estimates that each year in the United States, RSV causes 2.1 million outpatient visits in the population under 5 years of age, with 58,000–80,000 hospitalisations in children under 5 years of age [9]. In early childhood, it may increase the risk of recurrent wheezing and the development of asthma [10].

Among the population where vaccination against Streptococcus pneumoniae is common, 59.2% of hospitalised community-acquired pneumonia (CAP) cases were caused by viruses. In the Polish study, viral CAP was the leading confirmed aetiology, with Influenza and RSV being the major causes. Furthermore, viral CAP was confirmed in 41% of patients under 3 months of age, which is the highest risk group [11]. In addition, a multicentre Polish register found that 12% of SARS-CoV-2-infected children were diagnosed with pneumonia [12]. RSV, Influenza, and SARS-CoV-2 are among the leading viral causes of pneumonia in children, particularly in high-risk groups such as infants under 3 months of age. Since they share overlapping clinical manifestations (e.g., fever and cough) but differ in seasonality, age distribution, and risk of complications, direct comparison of these infections is warranted to improve clinical recognition, support differential diagnosis, and help optimise the use of diagnostic tests and therapeutic strategies. The purpose of this study was to comprehensively examine the symptomatology of these infections over a three-year period and to determine the predictors of aetiology.

## 2. Materials and Methods

This retrospective, observational study involved paediatric patients diagnosed with respiratory infections caused by RSV, Influenza, or SARS-CoV-2 over three consecutive seasons (2022/2023, 2023/2024, and 2024/2025). All participants were hospitalised at the Clinical Department of Paediatrics and Allergology, National Medical Institute of the Ministry of the Interior and Administration in Warsaw, Poland. Inclusion criteria were as follows: age < 18 years and hospitalisation with confirmed RSV, Influenza, or SARS-CoV-2 infection. Those above 18 years of age or managed only in a hospital emergency department or outpatient clinic were excluded from the study. The observation period ended with hospital discharge. RSV infection was confirmed by nasal swab using the RAPID-VIDITEST RSV test or a colorimetric immunochromatographic assay (test sensitivity 95% and specificity > 99%) [13]. SARS-CoV-2 virus was detected using the Abbott Panbio^TM^ rapid antigen test (test sensitivity 93% and specificity 99%) and by RT-PCR of genetic material using the Roche Cobas Liat analyser [14,15]. Influenza virus antigens were detected using an immunochromatographic test, a qualitative rapid test manufactured by HUMASIS (test specificity 100%).

The study analysed potential correlations between clinical symptoms and the type of viral infection, as well as the clinical presentation in different age groups (0–6 months, 7–24 months, and >24 months). Clinical symptoms, the disease course, and laboratory findings (e.g., CRP, leukocytosis, and oxygen saturation) were analysed for each patient. Fever was defined as a temperature of ≥38.6 °C, oxygen desaturation as a value < 96%, and leukocytosis was assessed relative to age-specific norms. Late hospital admission was defined as hospitalisation ≥ 4 days after symptom onset, prolonged hospitalisation as lasting ≥ 7 days, and lymphadenopathy was assessed during physical examination. Auscultatory findings were classified as single (limited to one location) or numerous (present in more than one location). Data on vaccination and prophylaxis status (including Influenza and COVID-19 vaccines, palivizumab/nirsevimab for RSV) were not collected in this study.

The study was approved by the Bioethics Committee of the National Medical Institute of the Ministry of the Interior and Administration in Warsaw, Poland approval no. 71/2025. Informed consent was not required for the retrospective analysis of anonymised medical records of hospitalised patients. The collected data were analysed using the χ^2^ test, the odds ratios (OR), and 95% confidence intervals (CI). *p* values < 0.05 were considered statistically significant. An odds ratio was considered not statistically significant if the lower bound of the 95% CI was ≤1, even when the OR itself exceeded 1. Multinomial multivariable logistic regression was performed to evaluate associations between clinical features and viral infections (with COVID-19 as the reference), as well as between age groups and viral infections (with children aged 0–6 months as the reference). All statistical calculations were performed using Microsoft 365 applications [Excel (version 16.74)] and R software (version 4.2.0; R Foundation for Statistical Computing, Vienna, Austria).

## 3. Results

Across all three seasons (2022/2023; 2023/2024; and 2024/2025), the total study population comprised 321 children: 176 (54.8%) boys. Patients were aged ≤ 17 years (median: 15 months, mean: 34 months). Children were divided into three age groups: 0–6 months, 7–24 months of age, and >24 months. Among the total, 129 (36%) patients tested positive for RSV, 110 (38%) for Influenza, and 82 (26%) for SARS-CoV-2. During the seasons, there were 121, 75, and 125 patients, respectively. The number of patients with viral infections (RSV, Influenza, and COVID-19) in each age group for every season is presented in Table 1. Table 2 presents the prevalence of symptoms (number and percentage) in RSV, Influenza, and COVID-19 across the 2022/2023, 2023/2024, and 2024/2025 seasons. Fever, cough, nasal discharge, and sore throat were the most common symptoms in Influenza. Cough, nasal discharge, and sore throat were often observed in COVID-19. Dyspnoea, lower oxygen saturation, cough, nasal discharge, and numerous auscultatory changes were observed most frequently in RSV-related hospitalisation. Comparisons of OR symptom frequency depending on the age of the child diagnosis are presented in Table 3, based on the univariable analysis.

In the univariable analysis, the χ^2^ test identified the following significant associations. A significant association was demonstrated between fever and RSV, Influenza, and COVID-19 infection (*p* < 0.05, χ^2^). Fever was more frequent in children with Influenza than in those with RSV (OR 4.77 [95%CI: 2.71–6.62]). The presence of fever in RSV and Influenza was significantly correlated with age (*p* < 0.05, χ^2^). Fever in Influenza and RSV was more common in older patients than in those aged 0–6 months. In Influenza, the odds of fever were significantly higher in children aged 7–24 months (OR 5.25, 95% CI 1.13–24.42) and >24 months (OR 3.94, 95% CI 1.16–13.4). Similarly, in RSV, fever was more frequent in children aged 7–24 months (OR 2.89, 95% CI 1.21–6.94) and >24 months (OR 4.68, 95% CI 1.76–12.39). Duration of fever was shown to be significantly correlated to infections caused by RSV, Influenza, and COVID-19 (*p* < 0.05, χ^2^). The odds of a longer fever were higher in Influenza than in COVID-19 (OR 3.86, 95% CI 1.83–8.16). Our analysis revealed a statistically significant relationship between cough (all types and non-productive) and RSV, Influenza, and COVID-19 infections (*p* < 0.05, χ^2^). Cough was more frequent in children with both RSV and Influenza than in those with COVID-19. In Influenza, the odds of cough were significantly higher (OR 2.18, 95% CI 1.17–4.06) and this was similar in RSV (OR 83.51, 95% CI 11.11–627.41). A higher incidence of non-productive cough was shown in Influenza (OR 2.96, 95% CI 1.5–5.86) and in COVID-19 (OR 5.08, 95% CI 2.02–12.79) compared to RSV. A strong correlation was identified between both nasal blockage and nasal discharge with infections due to RSV, Influenza, and COVID-19 (*p* < 0.05, χ^2^). Patients infected with RSV had a higher probability of having nasal discharge than those with COVID-19 (OR 8.51, 95% CI 3.65–19.82). Patients infected with RSV also had a higher risk of having nasal blockage than those with Influenza (OR 3.33, 95% CI 1.91–5.82). A clear statistical association was observed between dehydration, dyspnoea, oxygen desaturation, sore throat, enlarged lymph nodes, auscultatory abnormalities, the duration of hospitalisation, and infections with RSV, Influenza, and COVID-19 (*p* < 0.05, χ^2^). Dehydration was more common in Influenza than in COVID-19 (OR 2.41, 95%CI 1.26–4.6). Dyspnoea occurred more frequently in RSV infections than in COVID-19 (OR 7.51, 95%CI 3.46–16.32). In the case of Influenza, symptoms that required hospitalisation appeared later than in the case of SARS-CoV-2 infections (OR 2.45, 95%CI 1.25–4.79) and in the case of RSV compared to COVID-19 (OR 5.65, 95%CI 2.93–10.87). A higher incidence of oxygen desaturation (<96%) was observed in the RSV patient group compared to the SARS-CoV-2 patient group (OR 3.14, 95CI% 1.54–6.42]. Sore throat was more frequent in children with Influenza than in those with COVID-19 (OR 1.93, 95CI% 1.07–3.49). Children with RSV had a higher risk of developing auscultatory abnormalities than those with COVID-19 (OR 21.33, 95CI% 10.35–43.98). Enlarged lymph nodes were more common in Influenza than in COVID-19 (OR 4.2, 95CI% 1.75–10.13). The duration of hospitalisation was longer in RSV patients than in those with COVID-19 (OR 2.91, 95CI% 1.13–7.51]. The statistical analyses did not reveal statistically significant differences between patient groups in presence of chronic conditions, white blood cells, or CRP levels (*p* > 0.05, χ^2^).

Although many variables appeared to be significantly associated in the univariable analysis, this effect did not persist in the multivariable analysis, suggesting that the association is not independent of other factors, such as age or sex. Only two variables remained significant predictors of RSV infection: auscultatory abnormalities (OR 15.9, 95CI% 1.49–169) and hospital admission ≥ 4 days after symptom onset (OR 32.5, 95CI% 1.19–907).

A statistically significant association was observed in the univariable analysis between fever, loss of appetite, dyspnoea, fever persisting ≥ 4 days, comorbid chronic conditions, auscultatory abnormalities, elevated CRP, leukopenia, and leucocytosis in all three age groups: 0–6 months, 6–24 months, and >24 months in RSV (*p* < 0.05, χ^2^). In Influenza infections, several clinical manifestations—including fever, loss of appetite, dehydration, leucocytosis, and leukopenia —showed a statistically significant association across the age groups 0–6 months, 6–24 months, and >24 months (*p* < 0.05, χ^2^). Clinical features such as dyspnoea, sore throat, lymphadenopathy, comorbid chronic conditions, and elevated CRP were sign ificantly correlated with age group (0–6 months, 6–24 months, and >24 months of age) in SARS-CoV2 infections (*p* < 0.05, χ^2^).

In the multivariable analysis adjusted for sex, using children aged 0–6 months as the reference group, several factors were identified as distinguishing characteristics of specific age groups within each infection. In RSV infection, children aged 7–24 months were more likely to present with higher CRP levels (OR 1.06, 95CI% 1.003–1.13), reduced appetite (OR 6.7, 95CI% 1.62–27.67), and a longer duration of fever (OR 7.22, 95CI% 1.47–35.59), while in children older than 24 months, only longer duration of fever remained significant (OR 16.82, 95CI% 2.14–162.4). For Influenza, in the 7–24 month age group, reduced appetite was the only characteristic feature (OR 13.55, 95CI% 1.79–102.81). In COVID-19, children aged 7–24 months more frequently had higher CRP levels (OR 1.108, 95CI% 1.001–1.226) and chronic diseases (OR 7.59, 95CI% 1.115–51.64), whereas in those older than 24 months, only CRP was significant (OR 1.16, 95CI% 1.047–1.31).

## 4. Discussion

Acute respiratory infections are a common cause of outpatient visits. Most of these infections are of viral origin. These infected patients share many common symptoms, which makes it difficult to identify the aetiology of the infection. We therefore collected data on the course of the disease and observed whether infection with a given virus was more likely to cause a specific pattern. We found that over three infectious seasons, there was a consistent trend that children with more severe respiratory manifestations and hospital admission ≥ 4 days after symptom onset were infected with RSV. We noticed that age group also had an impact on the clinical course of the RSV disease: in the youngest children (<6 months of age), the main problems were shortness of breath and auscultatory abnormalities, while the oldest group of patients (>24 months of age) was characterised by the longer duration of fever. Influenza and SARS-CoV-2 infections were characterised by milder courses with predominant upper respiratory symptoms. For Influenza, reduced appetite was the most common predictor in the 7–24-month age group. In COVID-19, children > 6 months more frequently had higher CRP levels.

### 4.1. SARS-CoV-2

In our study, SARS-CoV-2 infection was the least frequent in 2024/2025 (17 children), higher in 2022/2023 (29 children), and the highest in 2023/2024 (36 children). The COVID-19 Hospitalisation Surveillance Network (COVID-NET) reported that SARS-CoV-2 activity stabilised at a relatively lower level in the 2023/2024 and 2024/2025 seasons after a prolonged period of elevated activity during the pandemic [16]. The lower incidence of COVID-19 in the 2022/2023 season compared to 2023/2024 was observed both in our study population and in Polish statistics reported by the Polish Chief Sanitary Inspectorate, where hospitalizations for the years 2022 to 2024 were reported as 4.78%, 8.5%, and 5.4% [17,18]. The 2022/23 season was dominated by earlier SARS-CoV-2 Omicron sub-variants (e.g., BA.1 and BA.2), which were highly contagious but milder in children. In contrast, in the 2023/24 season, new SARS-CoV-2 Omicron sub-variants (e.g., XBB.1.5) emerged that were more capable of escaping acquired immunity (after vaccination or previous infection), which could lead to more infections and hospitalisations [19,20].

The National Multicentre Database SARTSer-PED showed that COVID-19 has a mild course and typically presents with fever and respiratory symptoms, which is consistent with our results [1]. In the Chinese and Polish studies, the most common symptoms were fever and cough, followed by rhinitis, fatigue, headache, diarrhoea, and dyspnoea [5,21]. The occurrence of fever was similar, but presence of cough had higher frequency in our study population than these two studies. The increase in the incidence of cough is probably a result of the easing of pandemic restrictions, which has led to greater exposure to pathogens and increased air pollution. The Turkish study also outlines abdominal pain and rash, as frequent characteristic symptom of COVID-19 [22]. A prospective Polish study observed symptoms characteristic of younger children (<5 years old) and teenagers. Fever, rhinitis, diarrhoea, and rash were commonly observed in children younger than 5 years. Teenagers more often presented with weakness, headache, sore throat, anosmia, and muscle and chest pain [12]. In a previously mentioned study, shortness of breath was found to be significantly more common in adolescents than in younger children. Our analysis found no correlation between this characteristic and age groups. In addition, we observed that children > 6 months of age with a SARS-CoV-2 infection more frequently had elevated CRP levels. Elevated CRP is most probably caused by a more mature inflammatory response and a more severe course of the disease in contrast to younger infants.

### 4.2. Influenza

Influenza morbidity remained at a similar level in 2022/2023 (31 children) and 2023/2024 (28 children) with peak in the 2024/2025 (51 children) season. The ECDC (European Centre for Disease Prevention and Control) reported an earlier onset and peak of Influenza in the 2022/2023 season compared to the previous four seasons, while the 2023/2024 season was reported to have returned to pre-COVID-19 pandemic patterns of Influenza activity [6,23]. These are the most recent data available, as information from the 2024/2025 season has not yet been published. Influenza statistics reported by the Polish Chief Sanitary Inspectorate, Department of Prevention and Control of Infectious Diseases, presented the highest morbidity in the year 2023, followed by 2022, and 2024 [17,18]. The differences may result from the more limited study group, which mainly included children or adolescents, while the statistics of the Chief Sanitary Inspectorate (GIS) refer to the entire Polish population.

Influenza is typically described as an acute respiratory illness with a high temperature, which increases rapidly in the first 12 h of the illness, accompanied by the onset of general symptoms. The fever may provoke febrile convulsions in children under 2 years of age [24]. In our analysis, fever (i.e., ≥38.6 °C) was common in Influenza, especially in comparison to RSV- or SARS-CoV-2-related hospitalisations. Our finding is consistent with studies conducted in Turkey and Egypt [5,22]. Apart from fever, cough was the most prominent clinical feature of Influenza in the literature [25]. Pyrexia duration was longer in Influenza cases compared to RSV and COVID-19. Our findings are not consistent with those of Lin et al., who observed that the proportion of children with a fever lasting ≥ 5 days was 8.8% after the pandemic, whereas during the pandemic it was significantly higher, reaching 30% [26]. The most reasonable explanation is that the patients who were hospitalised in Poland were in a more serious condition than those in China. As a result of the higher proportion of febrile children, there was an increased risk of dehydration. Studies from Egypt showed that sore throats were frequently observed in patients with Influenza, while Chinese studies reported this significantly less often [26,27].

### 4.3. RSV

We observed that RSV affected the largest number of subjects in the 2022/2023 (61 children) and 2024/2025 (47 children) seasons. The ECDC reported return of RSV’s seasonal pattern in the seasons of 2022/2023 and 2023/2024 and an increase in RSV positivity. These are the most recent data available, as information from the 2024/2025 season has not yet been published. The report on infectious diseases and poisonings in Poland in 2024 showed that the incidence rate in 2024 increased more than threefold compared to 2023, with the hospitalisation rate remaining still at around 50%. This is consistent with our data, which also shows that approximately four times as many children were hospitalised in the 2024/2025 season. Polish data from 2022 were unavailable because reporting began in 2023.

Auscultatory abnormalities, rhinorrhoea, nasal blockage, later hospital admissions (>4 days from the onset of infection), and the longest hospital stay were characteristic of RSV infection. Analysis using multivariable logistic regression demonstrated independent predictors of RSV infection: auscultatory abnormalities or hospital admission ≥ 4 days after symptom onset. Furthermore, our results suggest that RSV causes more severe illness than Influenza, which was confirmed in previous papers [28]. More than half of RSV-hospitalised patients had dyspnoea, which reinforces the evidence provided by earlier study from Egypt [27]. In our analysis, the length of hospital stay was correlated with the aetiology of the infection with the longest stays being associated with RSV infection. Consistent with our findings, a publication from Israel reported that the mean length of hospital stay for RSV cases range from 3.75 to 6.1 days [29]. Fever (i.e., ≥38.6 °C) was observed in half of the patients, which is in contrast with the findings of Kandeel et al., where it occurred in the majority of patients [27]. The majority of studies show that younger children are more susceptible to severe lower respiratory tract infections [28]. Similar patterns have been documented in our analysis, the youngest age group faced an increased risk of numerous auscultatory changes and dyspnoea. Therefore, this age group should be considered a susceptible population and future interventions, including vaccinations, should be planned. In 2025, RSV vaccinations for pregnant women were been introduced in Poland, which should be considered a great preventive measure. The significant burden of disease and high risk of severity in young children infected with RSV makes it a widely studied area of interest. However, the standard of care does not differ between different respiratory infections, such as RSV, SARS-CoV-2, or Influenza, and remains mainly supportive, including fluids, antipyretics, and oxygen support as needed. With one exception, antiviral treatments for Influenza, such as neuraminidase inhibitors or the capsid-dependent endonuclease inhibitor (baloxavir), can be given to patients in the early stages of the disease [25]. Conversely, no specific antiviral treatment is currently available for RSV infection, although passive prophylaxis and monoclonal antibodies (palivizumab), are used in the group with an increased risk of severe RSV infection.

### 4.4. Limitations

The limitations of the study are as follows: it is a retrospective, single-centre study focusing on hospitalised patients. Therefore, the conclusions drawn from the study may not represent the epidemiology in the larger population. Furthermore, identifying the aetiology of respiratory infections is challenging, as upper respiratory tract (URT) samples can be contaminated and may correlate poorly with pathogens identified in the lower respiratory tract [30].

## 5. Conclusions

We identified that auscultatory abnormalities and hospital admission ≥ 4 days after symptom onset were independent predictors of RSV infection. Age also influenced the clinical course of RSV: children aged 7–24 months presented with higher CRP levels, reduced appetite, and a longer duration of fever, while those >24 months of age were primarily characterised by the longer fever duration. Influenza and SARS-CoV-2 infections were generally milder with predominant upper respiratory symptoms. In Influenza, reduced appetite was the most common predictor in children aged 7–24 months. In COVID-19, children older than 6 months more frequently had higher CRP levels. Overall, no single symptom reliably distinguishes the aetiology of viral respiratory infections. These age- and virus-specific patterns highlight the importance of continued surveillance and comparative research on major respiratory viruses in children, which remains crucial for clinical practice and public health response capabilities.

## Figures and Tables

**Table 1:** Number of paediatric patients with viral infections (RSV, Influenza, and COVID-19) by age group across the 2022/2023; 2023/2024; and 2024/2025 seasons.

	Season 2022/2023			Season 2023/2024			Season 2024/2025		
	COVID-19	RSV	Influenza	COVID-19	RSV	Influenza	COVID-19	RSV	Influenza
0–6 months of age	13	34	4	20	3	3	10	16	6
7–24 months of age	12	22	5	12	5	6	6	20	13
>24 months of age	4	5	22	4	3	19	1	21	32
Total	29	61	31	36	11	28	17	57	51

**Table 2:** Number and percentage of symptom prevalence in Influenza, RSV, COVID-19, and overall, during the 2022/2023, 2023/2024, and 2024/2025 seasons.

	Influenza = 110 N (%)	RSV = 129 N (%)	COVID-19 = 82 N (%)	Total = 321 N (%)
Fever (≥38.6 °C)	82 (74.55%)	46 (35.66%)	36 (43.9%)	164 (51.09%)
Cough	84 (76.36%)	124 (96.12%)	49 (59.76%)	257 (80.06%)
Non-productive cough	28 (25.45%)	28 (21.71%)	16 (19.51%)	72 (22.43%)
Nasal discharge	82 (74.55%)	118 (91.47%)	52 (63.41%)	252 (78.5%)
Nasal obstruction	27 (24.55%)	65 (50.39%)	22 (26.83%)	114 (35.51%)
Dehydration	44 (40%)	35 (27.13%)	18 (21.95%)	97 (30.22%)
Dyspnoea	9 (8.18%)	80 (62.02%)	17 (20.73%)	106 (33.02%)
Fever persisting ≥ 4 days	40 (36.36%)	24 (18.6%)	11 (13.41%)	75 (23.36%)
Hospital admission ≥ 4 days after symptom onset	41 (37.27%)	72 (55.81%)	16 (19.51%)	129 (40.19%)
Oxygen saturation < 96%	11 (10%)	45 (34.88%)	12 (14.63%)	68 (21.18%)
Comorbid chronic conditions	32 (29.09%)	24 (18.6%)	14 (17.07%)	70 (21.08%)
Sore throat	76 (69.09%)	60 (46.51%)	44 (53.66%)	180 (56.07%)
Auscultatory abnormalities	24 (21.82%)	106 (82.17%)	17 (20.73%)	147 (45.79%)
Lymphadenopathy	31 (28.18%)	9 (6.98%)	7 (8.54%)	47 (14.64%)
Hospitalisation lasting ≥ 7 days	6 (5.45%)	23 (17.83%)	6 (7.32%)	35 (10.9%)

**Table 3:** Occurrence of symptoms depending on the age of the child diagnosis, data aggregated from 2022/2023; 2023/2024; and 2024/2025 seasons (OR; [95%CI])—only the variables presented in the table reached statistical significance in the χ^2^ test (*p* < 0.05); significant OR values highlighted in blue.

	0–6 Months of Age	7–24 Months of Age	>24 Months of Age
**RSV**			
Fever ≥ 38.6 °C	1	2.89 [1.21–6.94]	4.68 [1.76–12.39]
Loss of appetite	1	5.79 [2.19–15.35]	1.51 [0.59–3.89]
Dyspnoea	7.38 [2.62–20.82]	3.07 [1.16–8.16]	1
Fever persisting ≥ 4 days	1	6.5 [1.71–24.77]	7.5 [1.82–30.92]
Comorbid chronic conditions	1	3.78 [1.23–11.62]	10.34 [3.14–34.01]
Auscultatory abnormalities	9.8 [1.91–50.24]	1.64 [0.55–4.92]	1
Elevated CRP	1	5.12 [1.84–14.31]	2.12 [0.85–5.3]
Leukopenia	0.62 [0.08–4.66]	10.8 [2.23–52.27]	1
Leucocytosis	3.38 [0.69–16.6]	4.2 [0.78–22.53]	1
**Influenza**			
Fever ≥ 38.6 °C	1	5.25 [1.13–24.42]	3.94 [1.16–13.4]
Loss of appetite	1	11.20 [2.16–58.13]	5.27 [1.52–18.25]
Dehydration	3.5 [0.64–19.24]	1	7.19 [1.97–26.24]
Leucocytosis	1.43 [0.26–7.82]	2.1 [0.54–8.12]	1
Leukopenia	-	1.1 [0.27–4.51]	1
**COVID-19**			
Dyspnoea	1	5.85 [1.42–24.04]	10.4 [1.78–60.62]
Sore throat	1	5.13 [1.84–14.29]	6.53 [1.2–35.48]
Lymphadenopathy	1	3 [0.26–34.68]	21 [1.87–236.03]
Comorbid chronic conditions	1	10.25 [2.05–51.26]	5.86 [0.7–48.68]
Elevated CRP	1	4.36 [1.16–16.37]	15.5 [2.74–87.74]

## Data Availability

The study is retrospective in nature and is based solely on the analysis of archival data previously collected as part of routine patient diagnostics and treatment. These data are analysed in a fully anonymized manner, making it impossible to identify the individuals involved. The study does not interfere in any way with patient care or affect their wellbeing. It also carries no risk for participants, as it does not require patient contact, involve additional medical procedures, or collect new personal data. There is no violation of patient rights, privacy, or medical ethics in the course of this study. According to Polish law, this study is not subject to the General Data Protection Regulation (GDPR), as no personal data are being processed within the meaning of Article 4(1) of the GDPR. Every patient in the ward is covered by the hospital’s general information clause regarding the processing of personal data in connection with the conduct of scientific research at PIM MSWiA in Warsaw.

## Abbreviations

The following abbreviations are used in this manuscript:

RSV	Respiratory Syncytial Virus
CAP	Community-acquired Pneumonia
ECDC	European Centre for Disease Prevention and Control
